# The Differential Contribution of the Innate Immune System to a Good Pathological Response in the Breast and Axillary Lymph Nodes Induced by Neoadjuvant Chemotherapy in Women with Large and Locally Advanced Breast Cancers

**DOI:** 10.1155/2017/1049023

**Published:** 2017-08-23

**Authors:** Viriya Kaewkangsadan, Chandan Verma, Jennifer M. Eremin, Gerard Cowley, Mohammad Ilyas, Sukchai Satthaporn, Oleg Eremin

**Affiliations:** ^1^Division of Gastrointestinal Surgery, Nottingham Digestive Diseases Centre, Faculty of Medicine and Health Sciences, University of Nottingham, E. Floor West Block, Queen's Medical Centre, Derby Road, Nottingham NG7 2UH, UK; ^2^Research & Development Department, Lincoln Breast Unit, Lincoln County Hospital, Greetwell Road, Lincoln LN2 5QY, UK; ^3^Department of Pathology, Path Links, Lincoln County Hospital, Greetwell Road, Lincoln LN2 5QY, UK; ^4^Academic Department of Pathology, Faculty of Medicine and Health Sciences, University of Nottingham, A Floor West Block, Queen's Medical Centre, Derby Road, Nottingham NG7 2UH, UK; ^5^Department of Surgery, Phramongkutklao Hospital and College of Medicine, 315 Rajavithi Road, Bangkok 10400, Thailand

## Abstract

The tumour microenvironment consists of malignant cells, stroma, and immune cells. The role of adaptive immunity in inducing a pathological complete response (pCR) in breast cancer with neoadjuvant chemotherapy (NAC) is well studied. The contribution of innate immunity, however, is poorly documented. Breast tumours and axillary lymph nodes (ALNs) from 33 women with large and locally advanced breast cancers (LLABCs) undergoing NAC were immunohistochemically assessed for tumour-infiltrating macrophages (TIMs: M1 and M2), neutrophils (TINs), and dendritic cells (TIDCs) using labelled antibodies and semiquantitative methods. Patients' blood neutrophils (*n* = 108), DCs (mDC1 and pDC), and their costimulatory molecules (*n* = 30) were also studied. Pathological results were classified as pCR, good (GPR) or poor (PRR). In breast and metastatic ALNs, high levels of CD163^+^ TIMs were significantly associated with a pCR. In blood, high levels of neutrophils were significantly associated with pCR in metastatic ALNs, whilst the % of mDC1 and pDC and expression of HLA-DR, mDC1 CD40, and CD83 were significantly reduced. NAC significantly reduced tumour DCs but increased blood DCs. PPRs to NAC had significantly reduced HLA-DR, CD40, and CD86 expression. Our study demonstrated novel findings documenting the differential but important contributions of innate immunity to pCRs in patients with LLABCs undergoing NAC.

## 1. Background

Anticancer immune mechanisms play an important role in the induction, development, and dissemination of malignant disease in man [1–4]. Both innate and adaptive immune cells have been documented in various human cancers (breast, gastrointestinal, urogenital, head and neck, and melanoma), and the presence of a prominent lymphocytic infiltrate is associated with a good clinical outcome [4–8]. In women with breast cancer undergoing neoadjuvant chemotherapy (NAC), the presence of a high level of tumour-infiltrating lymphocytes (TILs) has been shown to be an independent predictor of a complete pathological response (pCR) in the breast tumour [9–13]. The presence of TILs infiltrating axillary lymph node (ALN) metastases and their association with a pCR, however, is less well studied (Kaewkangsadan et al., 2016b submitted for publication).

The role of adaptive immunity (T effector and regulatory cells, T helper (Th), and suppressor cytokines) in women with breast cancer undergoing NAC has been investigated in primary breast tumours, but much less so in tumour-draining ALNs. Significant associations have been documented between high levels of infiltration by T effector cells (CD4^+^ and CD8^+^) and low levels of T regulatory cells (Tregs: FOXP3^+^ (forkhead box protein 3) and CTLA-4^+^ (cytotoxic T lymphocyte antigen-4)) and subsequent pCRs with NAC in primary breast tumours and ALN metastases [13–17]; (Kaewkangsadan et al., 2016b submitted for publication). The contribution from innate immunity (natural killer (NK) cells, macrophages, neutrophils, and dendritic cells (DCs)) to breast cancer cell death during NAC, however, is less well studied and poorly documented.

We have recently described the key role played by NK cells within primary breast tumours and tumour-draining ALNs. Our studies have shown a significant association between high levels of tumour-infiltrating CD56^+^ NK cells, in both the primary tumours and ALN metastases, and subsequent pCRs with NAC. These findings highlight the important contribution of innate immunity to immune-mediated tumour cell death in patients with breast cancer undergoing NAC.

Macrophages are terminally differentiated myeloid cells, closely related to DCs, and are resident in tissues. Human solid tumours recruit macrophages into their microenvironment. Breast cancers, in particular, contain a substantial number of macrophages [18]. Clinical and experimental evidence indicate that tumour-infiltrating macrophages (TIMs) play a major role in the initiation, progression, and dissemination of malignant disease [19]. Two main polarised phenotypes (M1 and M2) have been described. High levels of TIMs in most cancers, including breast cancer, are associated with a poor disease-free survival (DFS) and overall survival (OS) [20–23]. Heys et al. described high levels of the M1 CD68^+^ TIMs in breast cancers. [24]. Therefore, the association between M2 CD163^+^ TIMs and tumour response to NAC in both the breast and ALNs was examined.

Polymorphonuclear leukocytes (neutrophils) play a crucial role in dealing with invading pathogens and assist in wound healing. They release a range of toxic substances (reactive oxygen species and proteinases) to deal with invading microorganisms [25]. Increased levels of circulating neutrophils have been documented in patients with various solid cancers (breast, colon, pancreas, head and neck, and melanoma) in the absence of overt infection and inflammation. They have been shown to be immature, producing low levels of free radicals and showing impaired intracellular killing of pathogens [26]. Increased levels in blood in various solid cancers have been shown to be associated with poor clinical outcome [26]. Neutrophils are recruited into the tumour milieu by chemotactic stimuli from macrophages and tumour cells and have been described modifying tumour growth and invasiveness [27–29]. The presence of CD66b^+^ tumour-infiltrating neutrophils (TINs) has been shown to be associated with a reduced DFS and OS in human cancers (renal, head and neck, hepatic, and melanoma) [30–33]. There is a dearth of data on the significance of TINs in LLABCs and the interaction with NAC and possible tumour pCR. In this study, we have investigated the association between TINs and subsequent pCR with NAC in both the primary tumour and ALN metastases, and concurrently, neutrophils in the circulation.

Dendritic cells (DCs) are derived from haematopoietic progenitor cells in the bone marrow, monocytes being major precursor cells. They are crucial antigen-presenting cells, initiating and directing adaptive immune responses [34]. Various types have been categorised according to their morphological characteristics, phenotypic profiles, and in situ residence. Two main types, the large myeloid-derived DC (mDC) and the small plasmacytoid DC (pDC), upregulate the expression of coregulatory and costimulatory molecules (HLA-DR, CD40, CD80, and CD86) and the lymph node homing (LNH) molecules (CD197), enhancing adaptive immunity [35–37].

Various factors, including tumour stroma-released chemokines and cytokines (CCL19 and transforming growth factor-*β* (TGF-*β*)), hypoxia, and prostaglandins (PGE2), act as chemoattractants and induce DC recruitment into the tumour microenvironment [38]. DCs have been shown to infiltrate various types of human tumours [39]. In breast cancer, there is evidence that DCs in the tumour microenvironment are present in very low numbers and are poorly activated [40–42]. Moreover, dysfunctional DCs (suppressive and tolerogenic) have been identified in the circulation and tumour-draining ALNs in patients with operable breast cancer [38, 43]. Coventry et al. demonstrated a nonsignificant-enhanced 5-year survival rate in patients with breast cancer whose tumours had high levels of infiltration by CD1a^+^ DCs [44]. There is no published data on the significance and effect of circulating and TIDCs on pCR with NAC in breast cancer. We have investigated this in the blood, breast, and ALNs in the current study.

We have recently documented, in women with LLABCs undergoing NAC, that a significant reduction of both circulating and tumour-infiltrating Tregs (FOXP3^+^, CTLA-4^+^) and high levels of TILs and CD8^+^ T cells in the primary and ALN metastatic tumours were significantly associated with subsequent pCRs [13]; (Kaewkangsadan et al., 2016b submitted for publication). We also demonstrated that high CD8^+^ : FOXP3^+^ T cell ratios in the primary breast tumours and metastatic ALNs (tumour-free paracortex) were significantly associated with pCRs, highlighting the close and complex interrelationships between NAC and adaptive immunity. Furthermore, we recently described the important relationship between NK cells (blood, tumour, and ALNs), a key component of innate immunity, and NAC and pCR responses [45]. We have studied further the role of other components (TIMs, neutrophils, and DCs (blood and tumour)) of innate immunity in women with LLABCs undergoing NAC and their possible contribution to the pCRs that occur with chemotherapy and document our novel findings in this article.

## 2. Materials and Methods

### 2.1. Patients and Samples

Paraffin-embedded sections of breast tumours and tumour-draining ALNs from 33 women with large (L: ≥3 cm) and locally advanced breast cancers (LABCs: T3, 4; N1, 2; M0), enrolled in a study of NAC (108 patients enrolled between 2008 and 2011), were studied [46]. Histological diagnosis was established from ultrasound-guided core biopsies. To minimise tumour heterogeneity and sampling discrepancies, several core biopsies were obtained from each tumour. All tumours prior to NAC had a radiopaque coil inserted. Post-NAC, wire-guided removal of the residual “tumour” was carried out (in the case of breast conservation) if there was no clinical or radiological evidence of cancer. Operative specimens (wide local excision and mastectomy) had radiological confirmation of the presence of the coil to ensure accurate localisation and histopathological evaluation. Twenty-four patients had nodal metastases, and 9 patients were without nodal metastases; 20 out of 24 patients with nodal metastases had additional pre-NAC ultrasound-guided core needle biopsy samples of metastatic tumours in ALNs. Representative tissue sections were used for immunohistochemical (IHC) evaluation. All pre- and post-NAC specimens were discussed at a multidisciplinary meeting and a consensus reached about the pathological response and treatment options.

The NAC trial evaluated the effect of the addition of capecitabine (X) to docetaxel (T) preceded by adriamycin and cyclophosphamide (AC). Pathological responses were assessed in the excised surgical specimens after NAC. Established and previously published grading criteria were used to define histopathological responses in the breast [47, 48]. Good pathological responses were graded 5 (pCR, no residual invasive disease) and 4 (≥90% loss of invasive disease). Patients with grade 5 and 4 responses constituted the good pathological responders (GPRs). Poor pathological responses were graded as 3 (30–90% loss of invasive disease), 2 (<30% loss of invasive disease), and 1 (no loss of tumour cells). Patients with these responses constituted the PPR group. Pathological responses in metastatic tumours in ALNs were defined as pCR (grade 3: complete disappearance of tumour deposits or replacement by fibrosis in a previously histologically confirmed metastatic ALN); pathological partial response (grade 2: residual metastatic tumour deposits present with evidence of tumour destruction and replacement by fibrosis); and no pathological response (grade 1: metastatic tumour deposits remain with no evidence of fibrosis). Patient cases were randomly selected based on availability of tissue specimens and to ensure an even distribution between compared groups (pCR versus non-pCR).

The study was given approval by the Leicestershire, Northamptonshire & Rutland Research Ethics Committee 1: Reference number 07/H0406/260; Favourable Opinion 24/01/2008. All patients enrolled in the study gave informed consent to participate in and to publish the results of the study. The study registration is ISRCTN00407556.

### 2.2. Blood DC Phenotyping

Venous blood samples were collected before (*n* = 30) and following completion of NAC (*n* = 16). These blood samples were from the same patients whose tumour and nodal specimens were investigated immunohistochemically for CD1a^+^ DCs. Venous blood samples were also collected from 10 age-and sex-matched healthy female donors (HFDs) to establish normal values. Peripheral blood mononuclear cells (PBMCs) were isolated by centrifugation on Ficoll-Hypaque, washed and made up in RPMI with 10% foetal calf serum (FCS) (Sigma, UK) and antibiotics (TCM), and stored at −80°C for further analysis.

The mDC1^high^ (Lin1^−^, HLA-DR^+^, CD11c^+^, and CD1c^+^) and the pDC^high^ (Lin1^−^, HLA-DR^+^, CD11c^+^, and CD303^+^) subsets and their costimulatory molecules were documented in 2 × 10^6^ cells/100 *μ*l of PBMCs and stained for cell surface markers for 30 minutes (mins) with 5 *μ*l fluorescein isothiocyanate (FITC) anti-human LIN1, 5 *μ*l Texas red conjugate (ECD) anti-human HLA-DR, 5 *μ*l Pacific Blue anti-human CD11c, 5 *μ*l allophycocyanin (APC) Cy7 anti-human CD40, 5 *μ*l Pe-Cy7 anti-human CD80, 5 *μ*l APC anti-human CD86, 5 *μ*l Alexa 700 anti-human CD197, 2.5 *μ*l Percp-fluor-710 anti-human CD1c (BDCA-1) for mDCs^high^, and 5 *μ*l CD303-PE (BDCA-2) for pDCs^high^ which were added to the corresponding tubes and incubated for 30 mins at 4°C. The PBMC pellet was washed twice in phosphate-buffered saline. The PBMCs were then resuspended in 400 *μ*l of 0.5% paraformaldehyde fixative solution for flow cytometric analysis on a Beckman Coulter FC500.

Blood neutrophil counts (pre- and post-NAC) in 108 women with LLABC were collected and used in the analysis to evaluate association with NAC-induced pCR in the breast and tumour-draining ALNs.

### 2.3. Immunohistochemical (IHC) Assessment

Immunohistochemical assessments of immune cell subsets and expression of indoleamine 2,3-dioxygenase (IDO) and vascular endothelial growth factor (VEGF) were performed in 4 *μ*m tissue sections. Briefly, paraffin-embedded tissue sections were dewaxed and rehydrated using xylene and graded alcohol. Citrate buffer, pH 6.0, at 98°C was added for 20 mins for antigen retrieval. After serial blocking, the sections were incubated with the primary monoclonal antibody (MAb) against CD68 (Abcam, ab955, clone KP1), 1 : 300 dilution for 30 mins at room temperature (RT); MAb against CD163 (Abcam, ab74604, clone 10D6), prediluted for 30 mins at RT; MAb against CD1a (Dako, M3571, clone 010), 1 : 200 dilution for 15 mins at RT; MAb against CD66b (LS Bio, LS-B7134, clone 80H3), 10 *μ*g/ml for 30 mins at RT; MAb against IDO (Abcam, ab55305), 0.75 *μ*g/ml for 15 mins at RT; and MAb against VEGF (Dako, M7273, clone VG1), 1 : 50 dilution for 30 mins at RT. The Novolink™ polymer detection system, Leica RE7280-K with polymeric horseradish peroxidase- (HRP-) linker antibody conjugates and diaminobenzidine (DAB) chromogen, was used for enzyme-substrate labelling. Finally, the sections were counterstained with haematoxylin, dehydrated, and mounted in DPX mounting medium. Positive and negative staining controls were carried out with tonsil sections except for CD163 (liver sections) and IDO (normal colon sections). Negative staining controls were demonstrated by omitting the primary antibody. Positive and negative controls were simultaneously performed with every IHC staining run. All primary antibodies used in this study have been validated and certified by the commercial suppliers for IHC assessment of paraffin-embedded specimens.

### 2.4. Semiquantification of IHC Sections

Whole tissue sections were studied rather than microarrays (to minimise sampling bias). The majority of macrophages present in breast tumours were located along the border of tumour nests. Immunostaining for TIMs was evaluated along the tumour front (TF) over the whole section (7–10 view fields per section). This evaluation followed previously published studies documenting the level of TIMs [49–51]. Tumour tissue containing small areas of prominent infiltration of CD68^+^ or CD163^+^ cells, which was considerably higher than the average level of CD68^+^/CD163^+^ cells, was defined as hotspots (TF hotspot). All sections were evaluated at a distance away from areas of necrosis. The TF hotspots of the two view fields with the highest measurements at ×200 magnification were averaged out (CD68 or CD163 TF mean). The average infiltration (CD68 or CD163 TF mean) was semiquantitatively graded as no/weak (grade 1), moderate (grade 2), strong/robust (grade 3), and massive infiltration (grade 4). Tumours classified as 1 included totally negative specimens as well as specimens containing some scattered positively stained cells along the tumour margin. Tumours were classified as 2 when CD68 or CD163 staining was continuous along the tumour margin but did not extend from the tumour front for more than one cell layer on average. CD68 or CD163 staining that, on average, extended two to three cell layers from the tumour margin over the whole section was classified as 3; CD68 or CD163 staining extending more than three cell layers from the tumour margin in all fields was classified as 4. For statistical analysis, grades 1 and 2 were defined as low level of infiltration whereas grades 3 and 4 were defined as high level of infiltration [49–51].

To evaluate the presence and extent of CD1a^+^ and CD66b^+^ cells in the breast tumours, the average numbers of brown membrane-stained cells regardless of the intensity were counted in 5 high-power fields (400x HPFs). Positively stained cells in contact with tumour cells or within the tumour cell nests were defined as “intratumoural” whereas positively stained cells in the interstitial stroma surrounding tumour nests were defined as “peritumoural/stromal.” Evaluation of infiltrations in post-NAC specimens was undertaken on residual tumour nests, and in the case of pCR (complete disappearance of invasive tumour cells in the specimen), in the tumour bed. The latter was characterised histologically as a hyalinised, amorphous area with haemosiderin deposits [52, 53].

Positively stained macrophages (CD68^+^ and CD163^+^) in ALNs were quantified as the average % of all cells in 5 HPFs in nonmetastatic medullary areas of ALNs. The average number of cell counts in 5 HPFs in nonmetastatic areas with the greatest accumulations of positively stained cells on scanning at low magnification was determined for CD66b^+^ and CD1a^+^ cells. These quantitative evaluations followed the methodological scoring for documenting various immune cell subsets present in ALNs in patients with breast cancer [42, 54].

To evaluate the presence of IDO and VEGF, the semiquantitative H scoring system was employed using whole tissue sections. The H score was calculated by multiplying the % of positive cells (tumour and immune) by a factor representing the intensity of immune reactivity (1 for weak, 2 for moderate, and 3 for strong), giving a maximum score of 300. A score of <50 was considered negative, and a score of 50–100 was considered weakly positive (1+). A score of 101–200 was regarded as moderately positive (2+) and a score of 201–300 as strongly positive (3+). Negative and 1+ were considered as low expression whereas 2+ and 3+ were considered as high expression [55, 56].

All scored assessments were performed without the knowledge of the patients' clinical and pathological parameters. The pathological responses were assessed by a consultant breast pathologist. The scoring systems used in establishing the biological markers present in breast tumours and metastatic tumours in ALNs, and ALNs in this investigation, followed previously published studies documenting the scoring systems employed (described above).

### 2.5. Statistical Analysis

Statistical analyses were performed with the IBM SPSS statistics software, version 21 (SPSS Inc., Chicago, IL, USA). Where the data did not follow a normal distribution, a nonparametric test (Mann–Whitney *U* test (between two variables/groups)) was used to compare the groups based on pathological responses. The Pearson chi-square test was performed to compare the binomial data (negative/low versus high) on level of TIMs and expression of IDO/VEGF between groups. To evaluate and compare the related sample data between pre-NAC and post-NAC groups, as well as primary breast tumours and metastatic tumours in ALNs, the related sample Wilcoxon signed-rank test and related sample McNemar test were performed for comparing the number of cell counts and the expression of IDO/VEGF, respectively. A probability value (*p* value) of equal to or less than 0.05 (2 tailed) was considered statistically significant.

The sample size of this study was based on a cohort of patients from a previous study in which circulating Tregs were documented pre- and post-NAC [57]. A sample size of at least 7 in each group having an 80% power to detect a difference between two groups with *p* values of ≤0.05 (two sided) was calculated by assuming the common standard deviation of circulating blood Tregs as 0.5. As our findings are derived from several assays of different parameters, the sample size of at least 7 in each group may not be optimal for some of the tests. In addition, multiple hypothesis testing may lower the significance of our findings.

## 3. Results

### 3.1. Patient and Tumour Characteristics and Responses to NAC

The patient and tumour characteristics of the 33 patients studied are shown in Table A (Supplementary Table available online at https://doi.org/10.1155/2017/1049023). The responses to NAC and the tumour recurrence and survival at 4 years of follow-up are documented.

### 3.2. High Levels of Infiltration of Breast Tumours in LLABCs by CD68^+^ and CD163^+^ Macrophages and Subsequent PCR with NAC

**Figure 1:**
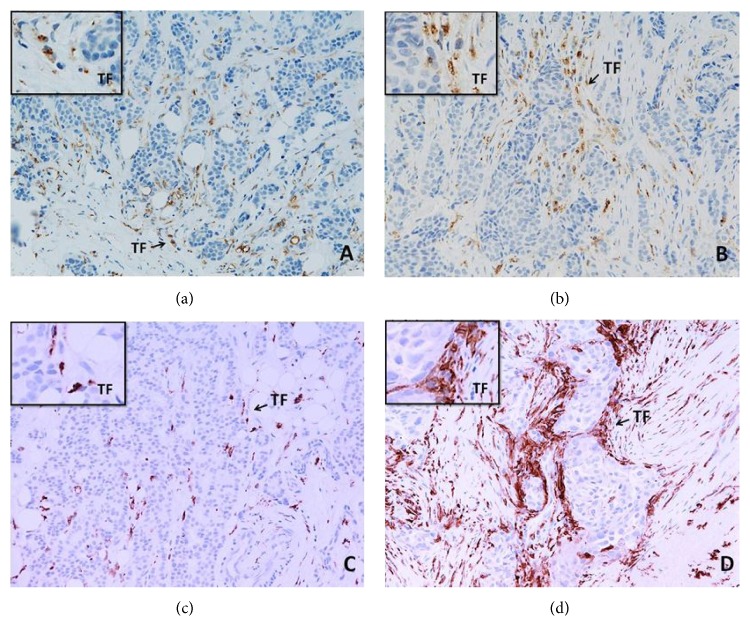
CD68^+^ (a, b) and CD163^+^ (c, d) macrophages in the sections of LLABCs, using IHC staining, at 200x magnification. Briefly, heat-mediated antigen retrieval was performed using citrate buffer, pH 6 (20 mins). The sections were then incubated with MAbs to CD68 (Abcam, ab955) at a 1 : 300 dilution for 30 mins at RT, MAbs to CD163 (Abcam, ab74604) at a prediluted concentration for 30 mins at RT. Polymeric HRP-linker antibody conjugate was used as secondary antibody. DAB chromogen was used to visualize the staining. The sections were counterstained with haematoxylin. (a, c) Low level of CD68^+^ and CD163^+^ macrophage infiltration; (b, d) high level of CD68^+^ and CD163^+^macrophage infiltration. Tumours were classified as low level of infiltration when the positively brown membrane-stained cells were scattered or continuous along the tumour margin but did not extend from the tumour front (TF) for more than one cell layer. Extension for two or more layers from the TF was classified as a high level of infiltration.

High levels of CD163^+^ TIMs in breast cancers were significantly associated with a GPR and pCR (*p* = 0.004, *p* = 0.008), respectively, following 8 cycles of NAC. There was, however, no significant association between CD68^+^ TIMs and GPR and pCR with NAC (Table 1).  Figure 1 illustrates the presence of high and low levels of infiltration by CD68^+^ (Figures 1(a) and 1(b)) and CD163^+^ macrophages (Figures 1(c) and 1(d)) in the primary tumours.

Eight cycles of NAC, on the other hand, did not alter the level of infiltration by CD68^+^ and CD163^+^ TIMs in post-NAC tumour specimens when compared with pre-NAC specimens (Table 2).

### 3.3. High Levels of Infiltration of ALN Metastases in LLABCs by CD163^+^ Macrophages and Subsequent PCR with NAC

**Figure 2:**
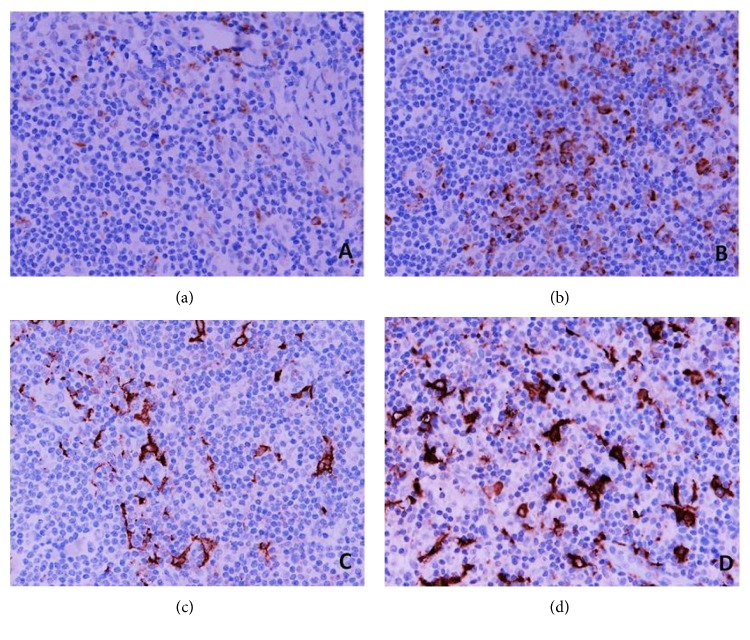
CD68^+^ (a, b) and CD163^+^ (c, d) macrophages in the sections of axillary lymph nodes (ALNs), using IHC staining, at 400x magnification. Briefly, heat-mediated antigen retrieval was performed using citrate buffer, pH 6 (20 mins). The sections were then incubated with MAbs to CD68 (Abcam, ab955) at a 1 : 300 dilution for 30 mins at RT, MAbs to CD163 (Abcam, ab74604) at a prediluted concentration for 30 mins at RT. Polymeric HRP-linker antibody conjugate was used as secondary antibody. DAB chromogen was used to visualize the staining. The sections were counterstained with haematoxylin. (a, c) Low percentage of CD68^+^ and CD163^+^ macrophages; (b, d) high percentage of CD68^+^ and CD163^+^macrophages. The positive brown membrane-stained cells in tumour-free medullary areas of ALNs were quantified as the average % of all cells (5 HPFs).

High levels of CD163^+^ TIMs in metastatic deposits in tumour-draining ALNs were significantly associated with a pCR (*p* = 0.003). As the levels of CD68^+^ TIMs in primary tumours showed no association with a pCR following NAC, we elected not to study this subset in the ALN metastases (Table 3).  Figure 2 illustrates CD68^+^ (Figures 2(a) and 2(b)) and CD163^+^ macrophages (Figures 2(c) and 2(d)) in the ALNs (tumour-free medullary areas).

Comparison of primary breast cancers with ALN metastases showed no significant differences in the levels of infiltration by CD163^+^ TIMs (Table 4).

### 3.4. Tumour Infiltration of Breast Tumours in LLABCs by CD1a^+^ DCs and CD66b^+^ Neutrophils Was Not Associated with a PCR following NAC

**Figure 3:**
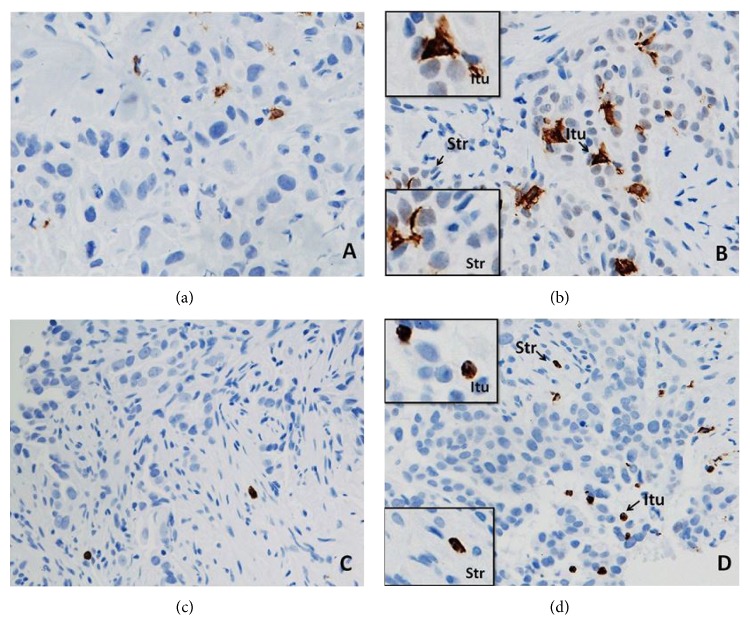
CD1a^+^ DCs (a, b) and CD66b^+^ neutrophils (c, d) in the sections of LLABCs, using IHC staining, at 400x magnification. Briefly, heat-mediated antigen retrieval was performed using citrate buffer, pH 6 (20 mins). The sections were then incubated with MAbs to CD1a (Dako, M3571) at a 1 : 200 dilution for 15 mins at RT, MAbs to CD66b (LSBio, LS-B7134) at a concentration of 10 *μ*g/ml for 30 mins at RT. Polymeric HRP-linker antibody conjugate was used as secondary antibody. DAB chromogen was used to visualize the staining. The sections were counterstained with haematoxylin. (a, c) Low level of CD1a^+^ DC and CD66b^+^ neutrophil infiltration; (b, d) high level of CD1a^+^ DC and CD66b^+^ neutrophil infiltration. The total number of brown membrane-stained cells, regardless of intensity, in contact with tumour cells or within tumour cell nests (Itu: intratumoural) and in the interstitial stroma (Str: stromal/peritumoural) in 5 HPFs was counted.

Neither good pathological responders nor those whose tumours had a pCR with NAC had a significant association with pre-NAC CD1a^+^ TIDCs or CD66b^+^ TINs. The infiltration was assessed both intratumourally and peritumourally (stroma) (Table 5). Figures 3(a) and 3(b) illustrate CD1a^+^ TIDCs and Figures 3(c) and 3(d) CD66b^+^ TINs in the tumour specimens examined. There was, however, a significant reduction in the level of intratumoural infiltration, but not peritumoural, by CD1a^+^ TIDCs with NAC (*p* = 0.001). There was, on the other hand, no intra- or peritumoural alteration in the level of CD66b^+^ TINs (Table 6).

### 3.5. No Significant Difference in the Levels of Macrophages (CD68^+^ and CD163^+^), Neutrophils, and DCs in ALNs (Metastatic versus Nonmetastatic) in Women with LLABCs Undergoing NAC

There was no significant difference in the level of macrophages (CD68^+^ and CD163^+^) in tumour-draining ALNs, comparing metastatic (tumour-free areas) with nonmetastatic, in women with LLABCs undergoing NAC (Table 7). Similarly, there was no significant difference in the levels of CD1a^+^ DCs or CD66b^+^ neutrophils (Table 7).

**Figure 4:**
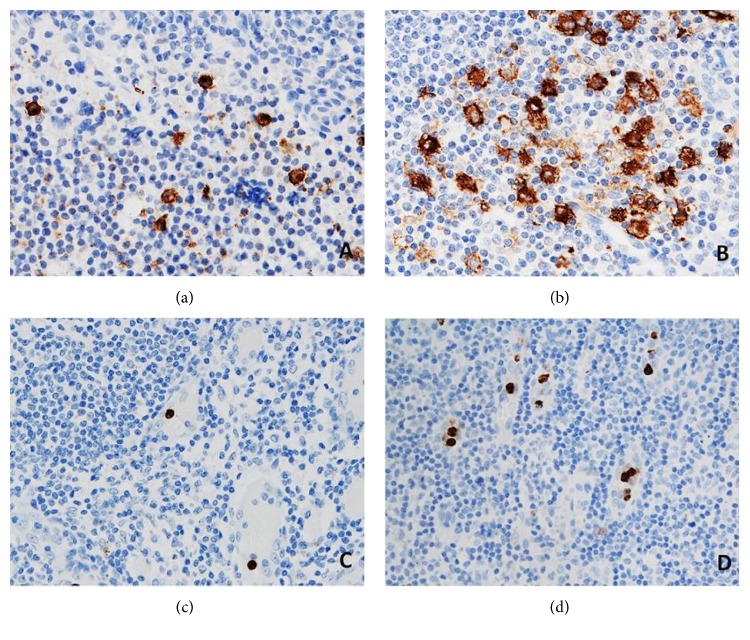
CD1a^+^ DCs (a, b) and CD66b^+^ neutrophils (c, d) in the sections of axillary lymph nodes (ALNs), using IHC staining, at 400x magnification. Briefly, heat-mediated antigen retrieval was performed using citrate buffer, pH 6 (20 mins). The sections were then incubated with MAbs to CD1a (Dako, M3571) at a 1 : 200 dilution for 15 mins at RT, MAbs to CD66b (LSBio, LS-B7134) at a concentration of 10 *μ*g/ml for 30 mins at RT. Polymeric HRP-linker antibody conjugate was used as secondary antibody. DAB chromogen was used to visualize the staining. The sections were counterstained with haematoxylin. (a, c) Low number of CD1a^+^ DCs and CD66b^+^ neutrophils; (b, d) high number of CD1a^+^ DCs and CD66b^+^ neutrophils. The average number of cell counts per HPF in tumour-free paracortical areas of ALNs with the greatest accumulation of the positive brown membrane-stained cells was quantified.

The presence of CD66b^+^ neutrophils and CD1a^+^ DCs in ALNs in tumour-free paracortical areas of ALNs is illustrated in Figures 4(c) and 4(d) and Figures 4(a) and 4(b), respectively.

### 3.6. Expression of VEGF and IDO in Women with LLABCs Undergoing NAC: CPR Significantly Associated with High Levels of VEGF in Pre-NAC Breast Cancers

High levels of expression of VEGF in primary breast cancers were significantly associated with a pCR (*p* = 0.018) following NAC. There was no association, however, with the expression of IDO (Table 8). In addition, there was no alteration of IDO and VEGF in the primary breast cancers following NAC (Table 9).

**Figure 5:**
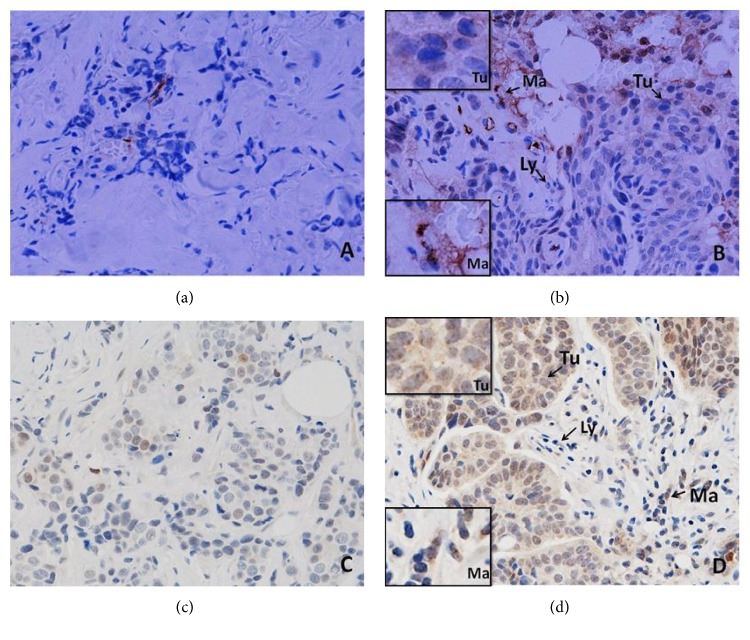
VEGF (a, b) and IDO (c, d) expression in the sections of LLABCs, using IHC staining, at 400x magnification. Briefly, heat-mediated antigen retrieval was performed using citrate buffer, pH 6 (20 mins). The sections were then incubated with MAbs to VEGF (Dako, M7273) at a 1 : 50 dilution for 30 mins at RT, MAbs to IDO (Abcam, ab55305) at a concentration of 0.75 *μ*g/ml for 15 mins at RT. Polymeric HRP-linker antibody conjugate was used as secondary antibody. DAB chromogen was used to visualize the staining. The sections were counterstained with haematoxylin. (a, c) Low level of expression; (b, d) high level of expression. The H score (% of positive cells (brown membrane/cytoplasmic-stained tumour and immune cells) × intensity of staining (1 to 3)) was used to assess the level of expression; low was ≤100 and high was >100. Scoring performed on a whole tissue section (7–10 HPFs); Tu: tumour, Ma: macrophage, and Ly: lymphocyte.

Figure 5 illustrates the expression of VEGF (Figures 5(a) and 5(b)) and IDO (Figures 5(c) and 5(d)), respectively, in primary breast cancers.

There was no difference in the expression of IDO and VEGF in ALNs, whether metastatic (tumour-free paracortical area) or nonmetastatic (data not shown).

**Figure 6:**
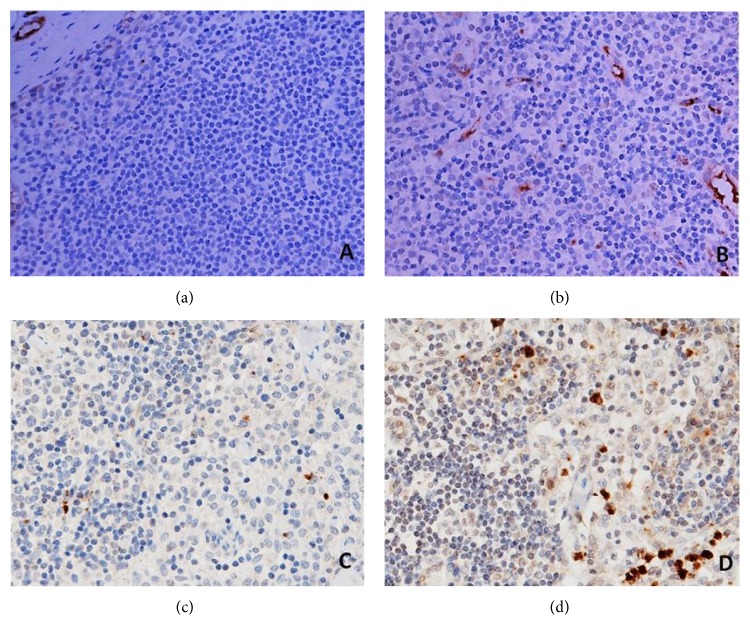
VEGF (a, b) and IDO (c, d) expression in the sections of axillary lymph nodes (ALNs), using IHC staining at 400x magnification. Briefly, heat-mediated antigen retrieval was performed using citrate buffer, pH 6 (20 mins). The sections were then incubated with MAbs to VEGF (Dako, M7273) at a 1 : 50 dilution for 30 mins at RT, MAbs to IDO (Abcam, ab55305) at a concentration of 0.75 *μ*g/ml for 15 mins at RT. Polymeric HRP-linker antibody conjugate was used as secondary antibody. DAB chromogen was used to visualize the staining. The sections were counterstained with haematoxylin. (a, c) Low level of expression; (b, d) high level of expression. The H score (% of positive cells (brown membrane/cytoplasmic-stained cells) × intensity of staining (1 to 3)) was used to assess the level of expression; low was ≤100 and high was >100. Scoring performed on nonmetastatic areas of a whole ALN section (7–10 HPFs).

Figure 6 illustrates the expression of VEGF (Figures 6(a) and 6(b)) and IDO (Figures 6(c) and 6(d)), respectively, in ALNs.

### 3.7. Significant Association between ER Status and Tumour Grade with CD163^+^ TIMs and PCR

High levels of CD163^+^ TIMs were significantly associated with ER status (*p* = 0.046) and tumour grade (*p* = 0.004). In addition, both parameters were significantly associated with tumour pCRs following NAC (*p* = 0.049, *p* = 0.010, resp.) (Table 10). Thus, ER-ve, high grade tumours were more likely to be infiltrated by TIMs and show a pCR with NAC. There was also a significant inverse association between pCR and recurrent disease (4-year follow-up) in the small group of patients (*n* = 33) studied (*p* = 0.001) (Table 10).

### 3.8. High Circulating Levels of PMN Neutrophils (Pre-NAC): Significant PCR in Metastatic ALNs

High levels of circulating PMN neutrophils pre-NAC in women with LLABCs (*n* = 108) were associated with a significant pCR in metastatic ALNs following 8 cycles of NAC. There were no comparable changes in the primary breast cancers, nor any significant associations with nodal status, DFS and OS (Table 11). There was also no significant correlation between blood and tumour-infiltrating CD66b^+^ neutrophils in patients following 8 cycles of NAC (data not shown). There was a significant reduction in the circulating levels of PMN neutrophils (*p* = 0.001) after NAC (Table 12). All patients were given granulocyte colony-stimulating factor (G-CSF) following randomisation.

### 3.9. Circulating Levels of DCs, mDC1s and pDCs, and Expression of Costimulatory and Lymph Node Homing Molecules in Women with LLABCs: Reduction in Blood Levels and Expression of Costimulatory Molecules

The % of DCs in the blood of women with LLABCs (*n* = 30) was significantly reduced (1.26 ± 0.20%) compared with healthy, age-matched female donors (HFDs (*n* = 9): 1.70 ± 0.30) (*p* = 0.034). The % of mDC1s^high^ was also significantly reduced compared with HFDs (0.10 ± 0.04% versus 0.15 ± 0.05%, *p* = 0.045), as was the % pDCs^high^ (0.03 ± 0.02% versus 0.06 ± 0.02%, *p* = 0.041) (Table 13).

The expression of the costimulatory molecule HLA-DR was significantly reduced in both the blood mDC1^+^ and pDC^+^ subsets, compared with HFDs (47.30 ± 8.00% versus 65.00 ± 3.10% (*p* = 0.045), 48.12 ± 9.50% versus 65.52 ± 5.00% (*p* = 0.002), resp.) (Table 13).

The expression of CD40 and CD83 was also depressed in mDC1s^high^, compared with HFDs (35.01 ± 8.00% versus 44.87 ± 6.50% (*p* = 0.011), 3.18 ± 0.40% versus 4.04 ± 0.30% (*p* = 0.047), resp.). The expression of CD80, CD86, and LNHR CD197 on the mDC1s^high^ were unaltered (Table 13). The levels of expression of these receptors on pDCs were inconsistent and difficult to document due to the small numbers of these cells in the blood, and apart from HLA-DR expression, are not shown.

### 3.10. Effect of NAC on the Circulating Levels of DCs in Women with LLABCs: Good Pathological Responders Have Significantly Increased Blood Levels following NAC

Before NAC, the % baseline levels of DCs in women who subsequently had a good pathological response with NAC (*n* = 9) were comparable with those that had a poor pathological response (*n* = 7) (1.64 ± 0.25 versus 1.29 ± 0.25) (Table 14). The % levels of blood DCs following 8 cycles of NAC and in those who had a good pathological response (pCR or ≥90% reduction in tumour mass) with NAC, however, were increased. This was even significantly higher than the levels documented in HFDs (3.24 ± 1.62 versus 1.70 ± 0.30, *p* = 0.024) (Table 14). There was no alteration in the post-NAC levels of circulating DCs in those patients whose tumours showed a poor pathological response (no response or <90% reduction in tumour mass) (Table 14).

### 3.11. Effect of NAC on the Circulating Levels and Expression of Costimulatory Molecules on mDC1s and pDCs: Blood Levels and Expression Lower in PPRs

After 8 cycles of NAC, those women whose breast cancers showed a PPR had significantly lower levels of blood mDC1s^high^ (*p* = 0.048) and pDCs^high^ (*p* = 0.017) and expression of HLA-DR (*p* = 0.001, *p* = 0.023, resp.), when compared with the blood levels of HFDs. Women whose tumours showed a GPR had no alterations in circulating levels (Table 15).

After 8 cycles of NAC, women whose breast cancers showed a PPR also had significantly lower levels of expression of mDC1 CD40 (*p* = 0.001) and CD86 (*p* = 0.001), compared with HFDs. These findings were not seen in women whose tumours had GPRs (Table 16).

Comparison of blood levels of mDC1 expression of costimulatory and LNH molecules between GPRs and PPRs showed a significant reduction in CD40, CD86, and CD197 expression on circulating mDC1s (*p* = 0.005, *p* = 0.004, and *p* = 0.003, resp.) following NAC in patients whose tumours had a PPR (Table 16).

These findings demonstrate the significant association between the reduced % (mDC1s and pDCs) and expression of HLA-DR (mDC1s and pDCs), CD40, and CD86 (mDC1s) and failure to achieve a GPR with NAC. Thus, women whose breast cancers failed to undergo a GPR with NAC showed significantly reduced, inactive, and immature DC subsets in the circulation.

## 4. Discussion

In the current study reported, the CD163^+^ TIMs (M2 macrophage phenotype) in both the primary breast cancer and metastatic ALNs were significantly associated with pCRs in malignant tissue following NAC. TIMs are derived from circulating monocytes and primitive bone marrow progenitors [58]. They are recruited into the tumour milieu by various chemokines (e.g., CCL2) and factors released by necrotic cells, in response to hypoxia and the cancer-associated fibroblasts [59]. Once embedded in the tumour microenvironment, the monocytes mature into the predominant M2 expressing CD163 (haemoglobin scavenger receptor) macrophages and are activated by Th 2 cytokines interleukin-4 (IL-4) and IL-10 [19, 60, 61]. The M2 phenotype favours tumour growth and metastatic spread through the promotion of angiogenesis, production of metalloproteinases, and inhibition of cytotoxic T lymphocytes (CTLs) by TGF-*β* and IL-10. TIMs also secrete arginase 1 in the tumour microenvironment, reducing L-arginine in situ and inhibiting CTL production and function [19, 60, 62–64]. Bingle et al., in a meta-analysis of various solid cancers, including breast cancer, showed that high levels of M2 TIMs were associated with a poor DFS and OS and this is supported by other, more recent publications [22, 65].

A pCR following NAC in breast cancer has been shown in some studies to be a surrogate marker for a good prognosis and survival [46, 66]. Cortazar et al. described a correlation between favourable outcome and pCR in breast cancer [67]. Our findings suggest that high levels of CD163^+^ TIMs in LLABCs are a predictor for a good pathological response and pCR but not necessarily for DFS and OS. In our study, there was a significant association between TIMs and tumour ER status and grade and between pCR and tumour ER status and grade. Highly proliferative, triple-negative breast cancers are significantly associated with high rates of pCR but have a poor DFS and OS [9, 46, 68]. Esserman et al. showed by studying breast cancer subsets that a pCR was a better predictor for a DFS [69]. A recent analysis revealed that although patients in whom there was a pCR in the tumour had a more favourable outcome, this did not always result in an improvement in OS [70].

M1 TIMs are activated by interferon-*γ* (IFN-*γ*) and bacterial products, produce proinflammatory cytokines, express IL-12, enhance adaptive immunity, and induce lysis of tumour cells [19–21]. In contrast to the presence of the M2 CD163^+^ TIMs, the M1CD68^+^ TIMs had no demonstrable association with a pCR. Heys et al. studied TIM subsets in breast cancer, using CD68 (general macrophage marker) expression of suppression of cytokine signalling (SOCS) 1 and SOCS 3 [24]. SOCS 1 (M2 phenotype) inhibits the proinflammatory signalling pathways downstream of IFN-*γ* and Toll-like receptor 4, whilst SOCS 3 (M1 phenotype) inhibits signal transduction and activation of transcription 3 (STAT 3) signalling. High levels of SOCS 3^+^ TIMs in LLABCs were associated with a pCR with NAC. There was, however, no association between SOCS 1^+^ TIMs and pathological response to NAC [24]. Thus, M1 and M2 TIM subsets, characterised by the expression of specific cellular functional markers, were associated with significant rates of pCR following NAC.

In sentinel lymph nodes (SLNs), Mansfield et al. found that the presence of sinusoidal CD163^+^ M2 macrophages was associated with a favourable nodal status in patients with breast cancer. They did not investigate TIMs in metastatic SLNs [54]. In our study, there was a significant association between the level of CD163^+^ TIMs in metastases in tumour-draining ALNs, prior to NAC and the subsequent pCR following 8 cycles of NAC. Our study, on the other hand, found no significant difference in the level of CD163^+^ macrophages in metastatic (tumour-free areas) versus nonmetastatic medullary areas in ALNs. The significant association between CD163^+^ TIMs in primary breast cancers and metastatic ALNs and pCRs with NAC has not been previously reported.

In patients with cancer, blood neutrophils tend to be immature and produce low levels of free radicals [26]. Increased levels of blood neutrophils have been documented in various tumours, including breast cancer, and shown to be associated with poor clinical outcomes [26]. Their association with pathological responses with NAC, however, has not been described. We did not demonstrate any association between blood neutrophils and poor clinical outcome, nor with a pCR in the primary tumour, in our cohort of 108 patients. We did, however, document a significant association between circulating levels of neutrophils, prior to NAC, and subsequent pCR in metastatic ALNs, a finding not previously described.

There were significantly reduced blood levels of PMN neutrophils following NAC, albeit all patients were given granulocyte colony-stimulating factor following randomisation.

Tumour entry of PMN neutrophils is induced by chemotactic molecules secreted by intratumoural TIMs and cancer cells [27, 28]. High levels of CD66b^+^ TINs have been found previously to be significantly associated with poor DFS and OS in a variety of nonbreast solid cancers [30–32]. In our study of women with LLABCs, CD66b^+^ TINs were not significantly associated with pathological responses to NAC. They were also resistant to NAC. The TINs were present in small numbers in the tumour microenvironment, and only a small cohort (*n* = 16) of patients was studied. A larger series is required to confirm these findings. There are, however, no published articles documenting their relevance in women with LLABCs undergoing NAC.

In tumour-draining metastatic ALNs, there was also no significant association between the level of infiltration by CD66b^+^ TINs and pCR with NAC. Moreover, there was no difference in the level of infiltration by CD66b^+^ neutrophils in the paracortical areas of metastatic (tumour-free areas) and nonmetastatic ALNs. Such findings have not previously been documented.

In our study, we investigated tumour-infiltrating CD1a^+^ DCs and concurrently circulating DCs, mDC1s^high^ and pDCs^high^. In solid cancers, evidence suggests that DCs in the tumour-microenvironment are present in small numbers, are immature, and poorly activated [41, 71, 72]. In patients with operable breast cancer, DCs in both ALNs and in the circulation have been shown to be dysfunctional [43]. In breast and ovarian cancer, moreover, TIDCs have been shown to be immunosuppressive, inhibiting the production of CTLs [38].

Although the presence of TIDCs has been documented to be associated with a better clinical outcome in a number of human solid cancers, this is not the case with CD1a^+^ TIDCs in breast cancer [44, 73–75]. There has, however, been no previously published study regarding CD1a^+^ TIDCs in LLABCs and the pathological responses with NAC. Our findings showed no significant association between the levels of CD1a^+^ TIDCs and pCR in either the primary tumours or metastatic ALNS. There is evidence that DCs in breast cancer (primary tumours and ALNs) are not only poorly activated and immature but may be also immunosuppressive, inhibiting the generation of CTLs and inducing T cell tolerance [38, 41, 43]. Tumour-induced inhibition of DC maturation and function is an important mechanism exploited by malignant cells to evade anticancer immunity [1, 76, 77]. Dumitriu et al. showed that DCs in the presence of lung carcinoma cells *in vitro* induced the secretion of TGF-*β* and enhanced the generation of CD4^+^CD25^+^ FOXP3^+^ Tregs [77]. We found that NAC significantly reduced the levels of intratumoural CD1a^+^ DCs although it had no effect on TIMs and TINs. NAC, by significantly reducing suppressor cells (Tregs and myeloid-derived suppressor cells) within the tumour microenvironment, may have contributed to the immune-associated tumour cell death [13, 57]. NAC, concurrently, had significant inhibitory effects on the circulating levels of DCs in the same cohort of patients.

The percentages of circulating levels of DCs, mDC1s and pDCs, were significantly reduced in women with LLABCs when compared with healthy females. In addition, the expression of HLA-DR, CD40, and CD83 molecules on the surface of mDC1s were also significantly reduced, as was HLA-DR expression of pDCs. The expression of the costimulatory molecules CD80/86 was unchanged compared with healthy females. We did not carry out any functional assays to ascertain specific effector functions such as efficacy in antigen presentation and secretion of cytokines. NAC, in particular anthracyclines and taxanes, is known to disrupt tumour cells, exposing expression of calreticulin and release of tumour-associated antigens (TAAs) [78, 79]. This drug combination was used in our NAC study [46].

There was a significant difference in the level of circulating DCs between those patients whose tumours showed a good pathological response (GPR; pCR or ≥90% loss of tumour cell mass) and those whose tumours had a poor pathological response (PPR; no or <90% loss of tumour cell mass) to 8 cycles of NAC. Following NAC, the blood DC levels in the GPR group were increased and were significantly higher than the levels documented in healthy females. There was also a significantly reduced % of circulating mDC1s^high^ and pDCs^high^ and reduced expression of HLA-DR, CD40, and CD83 on mDC1s^high^ and HLA-DR on pDCs^high^ in those patients whose tumours showed a PPR to NAC. In addition, post-NAC patients whose tumours had a GPR had a significantly increased expression of HLA-DR, CD40, CD86, and the LNHR CD197. These significant associations suggest a possible and important interaction between DCs and TAA presentation to naïve T cells and immune-induced tumour cell death during NAC. The findings documented, to the best of our knowledge, have not been published previously.

Many chemotherapeutic drugs produce short-lived inhibitory effects on innate and adaptive immune cells. Some (anthracyclines, taxanes, cyclophosphamide, capecitabine, and gemcitabine), however, can enhance or suppress specific aspects of the immune mechanism and activate immune-mediated tumour cell death, contributing to the good pathological responses documented in primary cancers and metastatic ALNs [13, 79–85], (Kaewkangsadan et al., 2016b submitted for publication). Chemotherapy, in particular anthracyclines, induces cancer cell stress and damage with resultant release of “danger signals” and release of immunogenic TAAs, enhancing the production of and tumour infiltration by TAA-specific CD8^+^ CTLs [78, 86, 87]. Capecitabine is converted to 5-fluorouracil in the body, increasing the expression of TAAs and inducing antibody-dependent cell-mediated cytotoxicity [88, 89]. Taxanes have been shown to increase circulating levels of INF-*γ*, IL-2, and IL-6, as well as enhancing NK cell cytotoxicity [80, 90]. Moreover, metronomic low dose cyclophosphamide has been documented to reduce the number and activity of FOXP3^+^ Tregs and to restore T effector and NK cell function in patients with disseminated disease [81].

Our study has provided further knowledge and understanding of the relevance and contribution of certain components of innate immunity to tumour cell death in both the primary breast cancer and metastases in tumour-draining ALNs in women with LLABCs undergoing NAC. High levels of TIMs (M2) appear to induce/enhance pCRs in primary and ALN metastatic breast cancers probably through their association with high tumour grade and negative ER status. High level of expression of VEGF and the resultant increased vascularity results in enhanced delivery of chemotherapeutic agents to the tumour cell milieu. Circulating levels of DCs and expression of HLA-DR and costimulatory molecules were significantly reduced in patients with LLABCs. This diminished number of activated DCs and thus decreased capacity to present TAAs (released by NAC and innate NK cells) to naïve adaptive T cells results in reduced generation of CTLs. This trend was significantly reversed in patients in whom NAC induced a pCR. These various immune mechanisms highlight the close and important interaction between innate and adaptive anticancer immunity.

## 5. Conclusion

There is a significant body of evidence documenting the important contribution by circulating and tumour-infiltrating T effector (CD4^+^ and CD8^+^) and regulatory (FOXP3^+^ and CTLA-4^+^) cells and NK cells in immune-mediated breast cancer cell death in women with LLABCs undergoing NAC. Our novel findings have further increased our knowledge and documented the important contribution of innate immunity to tumour cell death in women with LLABCs undergoing NAC. The significant associations between the beneficial pathological responses (GPR and pCR) in the tumour microenvironment (primary and ALN metastases) and key innate immune cells (CD163^+^ TIMs, circulating PMN neutrophils, and DCs/subsets) complement our findings with NK cells and are an important contribution to the understanding of putative anticancer immune responses in NAC, resulting in immune-mediated tumour cell death.

## Supplementary Material

Table A. Patient and Tumour Characteristics, Responses to Neoadjuvant Chemotherapy (n=33).

## Figures and Tables

**Table 1:** Analyses of tumour-infiltrating CD68^+^ and CD163^+^ macrophages in the breast tumours in women with LLABCs and subsequent PCR following NAC.

Macrophages	Groups	Pre-NAC			
		Low infiltration (*n*)	High infiltration (*n*)	Pearson chi-square value (GPR versus PPR, PCR versus non-PCR)	*p* value
CD68^+^ (*n* = 16)	Good pathological response (GPR, *n* = 9)	5	4	1.667	0.197
	Poor pathological response (PPR, *n* = 7)	6	1		
	Pathological complete response (PCR, *n* = 6)	3	3	1.571	0.210
	Nonpathological complete response (non-PCR, *n* = 10)	8	2		

CD163^+^ (*n* = 33)	Good pathological response (GPR, *n* = 21)	5	16	8.192	0.004^∗^
	Poor pathological response (PPR, *n* = 12)	9	3		
	Pathological complete response (PCR, *n* = 16)	3	13	7.127	0.008^∗^
	Nonpathological complete response (non-PCR, *n* = 17)	11	6		

LLABCs: large and locally advanced breast cancers; NAC: neoadjuvant chemotherapy; ^∗^statistically significant.

**Table 2:** Alteration of tumour-infiltrating CD68^+^ and CD163^+^ macrophages in the breast tumours in women with LLABCs undergoing NAC.

Macrophages	Groups		Post-NAC		*p* value^(3)^ (pre- versus post-NAC)
			Low infiltration (*n*)	High infiltration (*n*)	
CD68^+^ (*n* = 16)	Pre-NAC	Low infiltration (*n*)	10	1	0.375
		High infiltration (*n*)	4	1	

CD163^+^ (*n* = 16)	Pre-NAC	Low infiltration (*n*)	4	2	0.289
		High infiltration (*n*)	6	4	

LLABCs: large and locally advanced breast cancers; NAC: neoadjuvant chemotherapy; ^(3)^related sample McNemar test.

**Table 3:** Analyses of CD163^+^ macrophages in metastatic tumours in ALNs in women with LLABCs and subsequent PCR following NAC.

	Groups	Pre-NAC			
		Low infiltration (*n*)	High infiltration (*n*)	Pearson chi-square value (PCR versus non-PCR)	*p* value
CD163^+^ macrophages (*n* = 20)	Pathological complete response (PCR, *n* = 9)	0	9	8.811	0.003^∗^
	Nonpathological complete response (non-PCR, *n* = 11)	7	4		

ALNs: axillary lymph nodes; LLABCs: large and locally advanced breast cancers; NAC: neoadjuvant chemotherapy; ^∗^statistically significant.

**Table 4:** Comparison of CD163^+^ macrophages between primary breast tumours and ALN metastatic tumours in women with LLABCs.

Groups			Metastatic tumours in ALNs		*p* value^(3)^ (primary versus metastases)
			Low infiltration (*n*)	High infiltration (*n*)	
CD163^+^ macrophages	Primary tumours in breast	Low infiltration (*n*)	6	2	1.000
		High infiltration (*n*)	1	11	

ALN: axillary lymph node; LLABCs: large and locally advanced breast cancers; ^(3)^related sample McNemar test.

**Table 5:** Analyses of tumour-infiltrating CD1a^+^ dendritic cells and CD66b^+^ neutrophils in the breast tumours in women with LLABCs and subsequent PCR following NAC.

Cell subsets	Groups	Pre-NAC intratumoural median (range)^(3)^	*p* value^(3)^ (GPR versus PRR, PCR versus non-PCR)	Pre-NAC peritumoural median (range)^(3)^	*p* value^(4)^ (GPR versus PRR, PCR versus non-PCR)
CD1a^+^ (*n* = 16)	Good pathological response (GPR, *n* = 9)	3 (1–104)	0.837	1 (1–16)	0.837
	Poor pathological response (PPR, *n* = 7)	11 (0–63)		2 (0–11)	
	Pathological complete response (PCR, *n* = 6)	3 (1–104)	0.713	1.5 (1–16)	0.492
	Nonpathological complete response (non-PCR, *n* = 10)	4 (0–63)		1.5 (0–11)	

CD66b^+^ (*n* = 16)	Good pathological response (GPR, *n* = 9)	2 (0–53)	0.174	2 (0–71)	0.408
	Poor pathological response (PPR, *n* = 7)	1 (0–3)		1 (0–2)	
	Pathological complete response (PCR, *n* = 6)	3 (0–53)	0.181	5 (0–71)	0.118
	Nonpathological complete response (non-PCR, *n* = 10)	1 (0–3)		1 (0–2)	

LLABCs: large and locally advanced breast cancers; NAC: neoadjuvant chemotherapy; ^(3)^total cell count per 5 high-power fields (core biopsies of breast cancers); ^(4)^Mann–Whitney *U* test.

**Table 6:** Alteration of tumour-infiltrating CD1a^+^ dendritic cells and CD66b^+^ neutrophils in breast tumours in women with LLABCs undergoing NAC.

Groups		Pre-NAC median (range)^(3)^	Post-NAC median (range)^(3)^	*p* value^(4)^ (pre- versus post-NAC)
CD1a^+^	Intratumoural infiltration (*n* = 16)	3.5 (0–104)	0 (0–2)	0.001^∗^
	Peritumoural infiltration (*n* = 16)	1.5 (0–16)	1 (0–7)	0.184

CD66b^+^	Intratumoural infiltration (*n* = 16)	1 (0–53)	4.5 (0–50)	0.125
	Peritumoural infiltration (*n* = 16)	1.5 (0–71)	2.5 (0–82)	0.470

LLABCs: large and locally advanced breast cancers; NAC: neoadjuvant chemotherapy; ^(3)^cell count in 400x HPF; ^(4)^Wilcoxon signed-rank test; ^∗^statistically significant.

**Table 7:** Analyses of immune cell subsets in ALNs in women with LLABCs undergoing NAC.

Immune cell subsets	Groups	ALN median (range)^(4)^	*p* value^(5)^
CD68^+^ macrophages (*n* = 16)	Nonmetastatic ALNs (*n* = 9)	25.0 (14.8–34.0)	0.918
	Metastatic ALNs (*n* = 7)	29.0 (13.8–33.0)	
CD163^+^ macrophages (*n* = 33)	Nonmetastatic ALNs (*n* = 9)	21.0 (16.0–29.0)	1.000
	Metastatic ALNs (*n* = 24)	23.0 (10.0–33.0)	
CD1a^+^ DCs (*n* = 16)	Nonmetastatic ALNs (*n* = 9)	12.8 (0.8–62.0)	0.536
	Metastatic ALNs (*n* = 7)	23.8 (6.6–67.0)	
CD66b^+^ PMNs (*n* = 16)	Nonmetastatic ALNs (*n* = 9)	5.2 (0.6–94.0)	0.837
	Metastatic ALNs (*n* = 7)	8.4 (1.0–163.0)	

ALNs: axillary lymph nodes; LLABCs: large and locally advanced breast cancers; NAC: neoadjuvant chemotherapy; ^(4)^average percentage of positively stained cells out of all the lymphoid cells in the ALN sections examined for CD68^+^ and CD163^+^ macrophages, average cell count of positively stained cells per 400x high-power field in the ALN sections examined for CD1a^+^ DCs and CD66b^+^ PMNs; ^(5)^Mann–Whitney *U* test; DCs: dendritic cells; PMNs: polymorphonuclear leukocytes.

**Table 8:** Analyses of VEGF and IDO expression in the breast tumours in women with LLABCs (pre-NAC and post-NAC) and association with a PCR.

VEGF/IDO (*n* = 16)	Groups	Pre-NAC				Post-NAC			
		Low/negative expression (*n*)	High expression (*n*)	Pearson chi-square value (GPR versus PPR, PCR versus non-PCR)	*p* value	Low/negative expression (*n*)	High expression (*n*)	Pearson chi-square value (GPR versus PPR, PCR versus non-PCR)	*p* value
VEGF	Good pathological response (GPR, *n* = 9)	5	4	1.667	0.197	7	2	0.780	0.377
	Poor pathological response (PPR, *n* = 7)	6	1			4	3		
	Pathological complete response (PCR, *n* = 6)	2	4	5.605	0.018^∗^	5	1	0.950	0.330
	Nonpathological complete response (non-PCR, *n* = 10)	9	1			6	4		

IDO	Good pathological response (GPR, *n* = 9)	6	3	0.042	0.838	6	3	0.152	0.696
	Poor pathological response (PPR, *n* = 7)	5	2			4	3		
	Pathological complete response (PCR, *n* = 6)	3	3	1.571	0.210	4	2	0.071	0.790
	Nonpathological complete response (non-PCR, *n* = 10)	8	2			6	4		

VEGF: vascular endothelial growth factor; IDO: indoleamine 2,3-dioxygenase; LLABCs: large and locally advanced breast cancers; NAC: neoadjuvant chemotherapy; ^∗^statistically significant.

**Table 9:** No alteration of expression of IDO and VEGF in the breast tumours in women with LLABCs undergoing NAC.

VEGF/IDO (*n* = 16)	Groups		Post-NAC		*p* value^(5)^
			Low/negative expression (*n*)	High expression (*n*)	
VEGF	Pre-NAC	Low/negative expression (*n*)	8	3	1.000
		High expression (*n*)	3	2	

IDO	Pre-NAC	Low/negative expression (*n*)	8	3	1.000
		High expression (*n*)	2	3	

IDO: indoleamine 2,3-dioxygenase; VEGF: vascular endothelial growth factor; LLABCs: large and locally advanced breast cancers; NAC: neoadjuvant chemotherapy; ^(5)^related sample McNemar Test.

**Table 10:** Clinical and pathological parameters of patients (*n* = 33) studied and the Association of pre-NAC tumour-infiltrating CD163^+^ macrophages (TIMs) and pathological complete response (PCR).

Groups	TIMs				PCR			
	Low infiltration (*n*)	High infiltration (*n*)	Pearson chi-square value	*p* value	Non-PCR (*n*)	PCR (*n*)	Pearson chi-square value	*p* value
Age (years)								
<50	6	8	0.002	0.966	8	6	0.308	0.579
≥50	8	11			9	10		
BMI (kg/m^2^)								
≤30	10	10	1.193	0.275	11	9	0.247	0.619
>30	4	9			6	7		
Menopausal status								
Pre	5	11	1.588	0.208	8	8	0.029	0.866
Post	9	8			9	8		
Tumour size								
<40 mm	8	10	0.066	0.797	9	9	0.036	0.849
≥40 mm	6	9			8	7		
Nodal status								
Negative	5	5	0.337	0.561	5	5	0.013	0.909
Positive	9	14			12	11		
Tumour grade								
1 (low)	1	1	11.270	0.004^∗^	2	0	9.303	0.010^∗^
2 (moderate)	10	3			10	3		
3 (high)	3	15			5	13		
Oestrogen receptor								
Negative	2	9	3.970	0.046^∗^	3	8	3.882	0.049^∗^
Positive	12	10			14	8		
HER-2 receptor								
Negative	10	13	0.035	0.853	13	10	0.762	0.383
Positive	4	6			4	6		
NAC regimen								
AC-TX	6	10	0.308	0.579	6	10	2.443	0.118
AC-T	8	9			11	6		
Recurrent disease^(4)^								
No	8	14	0.992	0.319	7	15	10.252	0.001^∗^
Yes	6	5			10	1		
Death^(4)^								
No	11	16	0.172	0.678	12	15	2.972	0.085
Yes	3	3			5	1		

NAC: neoadjuvant chemotherapy; BMI: body mass index (≤30: nonobese, >30: obese); AC-TX: doxorubicin, cyclophosphamide, taxotere, and xeloda® (capecitabine), respectively; ^(4)^4-year follow-up; ^∗^statistically significant.

**Table 11:** Analyses of blood PMN neutrophils in women with LLABC and specific clinical and pathological parameters.

Groups		Pre-NAC median (range)^(3)^	*p* value^(4)^ (GPR versus PRR, PCR versus non-PCR)	Post-NAC median (range)^(3)^	*p* value^(4)^ (GPR versus PRR, PCR versus non-PCR)
Blood PMNs	Good pathological response (GPR, *n* = 52)	4.13 (2.15–11.30)	0.796	3.05 (1.40–6.75)	0.134
	Poor pathological response (PPR, *n* = 56)	4.07 (1.80–10.10)		3.47 (0.03–8.73)	
	Pathological complete response (PCR, *n* = 29)	4.50 (2.15–11.30)	0.381	3.10 (1.40–6.75)	0.755
	Nonpathological complete response (non-PCR, *n* = 79)	3.94 (1.80–10.10)		3.26 (0.03–8.73)	
	Nodal metastasis (*n* = 56)	3.94 (1.80–10.10)	0.634	3.11 (0.03–8.26)	0.337
	No nodal metastasis (*n* = 52)	4.16 (2.15–11.30)		3.36 (1.54–8.73)	
	Nodal pCR (*n* = 16)	5.43 (2.84–10.10)	0.002^∗^	3.20 (1.40–8.26)	0.892
	No nodal pCR (*n* = 40)	3.65 (1.80–9.17)		3.05 (0.03–6.37)	
	Recurrent disease^(5)^ (*n* = 23)	4.02 (1.80–8.70)	0.612	3.19 (0.03–5.80)	0.878
	No recurrent disease (*n* = 85)	4.10 (2.15–11.30)		3.23 (1.33–8.73)	
	Death^(5)^ (*n* = 17)	3.87 (2.23–6.64)	0.276	3.00 (0.03–5.42)	0.360
	Survive (*n* = 91)	4.11 (1.80–11.30)		3.23 (1.33–8.73)	

LLABC: large and locally advanced breast cancers; NAC: neoadjuvant chemotherapy; ^(3)^×10^9^ cells/litre; ^(4)^Mann–Whitney *U* test; ^(5)^4-year follow-up; ^∗^statistically significant.

**Table 12:** Alteration of blood PMN neutrophils in women with LLABCs undergoing NAC.

Group	Pre-NAC median (range)^(3)^	Post-NAC median (range)^(3)^	*p* value^(4)^ (pre- versus post-NAC)
Blood PMNs (*n* = 108)	4.09 (1.80–11.30)	3.20 (0.03–8.73)	<0.001^∗^

LLABCs: large and locally advanced breast cancers; NAC: neoadjuvant chemotherapy; ^(3)^×10^9^ cells/litre; ^(4)^Wilcoxon signed-rank test; ^∗^statistically significant.

**Table 13:** Percentage of DC subsets and expression of costimulatory and LNH molecules in the circulation of women with LLABCs.

mDC1s and pDCs	% of subsets and costimulatory molecules in women with LLABCs (*n* = 30)	% of subsets and costimulatory molecules in HFDs (*n* = 10)	*p* value
Lin1^−^, HLA-DR^+^ DCs	1.26 ± 0.20	1.70 ± 0.30	0.034^∗^
mDC1^high^ (Lin1^−^, HLA- DR^+^, CD11c^+^CD1c^+^)	0.10 ± 0.04	0.15 ± 0.05	0.045^∗^
pDC^high^ (Lin1^−^, HLA-DR^+^, CD11c^+^CD303^+^)	0.03 ± 0.02	0.06 ± 0.02	0.041^∗^
mDC1 HLA-DR*^+^*	47.30 ± 8.00	65.00 ± 3.10	0.045^∗^
pDC HLA-DR*^+^*	48.12 ± 9.50	65.52 ± 5.00	0.002^∗^
mDC1 CD40*^+^*	35.01 ± 8.00	44.87 ± 6.50	0.011^∗^
mDC1 CD80*^+^*	1.40 ± 0.60	1.87 ± 0.35	NS
mDC1 CD83*^+^*	3.18 ± 0.40	4.04 ± 0.30	0.047^∗^
mDC1 CD86*^+^*	28.56 ± 3.00	34.07 ± 8.00	NS
mDC1 CD197*^+^*	20.35 ± 4.50	24.68 ± 9.00	NS

LNH: lymph node homing; LLABCs: large and locally advanced breast cancers; mDC1: myeloid dendritic cell; pDC: plasmacytoid dendritic cell; HFDs: healthy female donors; NS: nonsignificant; ^∗^statistically significant.

**Table 14:** Effect of NAC on blood DC levels in women with LLABCs.

Pathological responders	DC baseline levels (%)
	*p* value

GPRs + PPRs	1.34 ± 0.30
GPRs + PPRs versus HFDs	NS
GPRs	1.64 ± 0.25
GPRs versus HFD	NS
PPRs	1.29 ± 0.25
PPRs versus HFDs	NS

Pathological responders	DC post-NAC levels (%)
	*p* value

GPRs	3.24 ± 1.62
GPRs versus HFDs	0.024^∗^
PPRs	2.17 ± 0.5
PPRs versus HFDs	NS

NAC: neoadjuvant chemotherapy; LLABCs: large and locally advanced breast cancers; GPRs: good pathological responders (pCR or ≥90% reduction of invasive disease); PPRs: poor pathological responders (no response or <90% reduction of invasive disease); HFDs: healthy female donors; NS: nonsignificant; ^∗^statistically significant.

**Table 15:** Percentage of DC subsets and expression of HLA-DR in the blood of women with LLABC undergoing NAC. Baseline (B) levels in LLABCs versus completion of chemotherapy (CC) levels in different responders.

Study group comparisons		mDC1	mDC1	pDC	pDC
			HLA-DR		HLA-DR
Good pathological responders (GPRs: *n* = 9)	B (%)	0.12 ± 0.04	36.07 ± 6.00	0.02 ± 0.02	50.86 ± 10.5
	CC (%)	0.10 ± 0.04	46.33 ± 8.00	0.05 ± 0.02	58.05 ± 12.00
	B versus CC (*p* value)	NS	NS	NS	NS
	CC versus HFDs (*p* value)	NS	NS	NS	NS

Poor pathological responders (PPRs: *n* = 7)	B (%)	0.08 ± 0.03	31.29 ± 6.00	0.02 ± 0.02	53.21 ± 10.00
	CC (%)	0.05 ± 0.03	29.83 ± 5.00	0.03 ± 0.02	49.97 ± 6.00
	B versus CC (*p* value)	NS	NS	NS	NS
	CC versus HFDs (*p* value)	0.048^∗^	0.001^∗^	0.017^∗^	0.023^∗^

Post-NAC GPR versus PPR	GPR CC versus PPR CC (*p* value)	NS	0.041^∗^	NS	NS

LLABCs: large and locally advanced breast cancers; NAC: neoadjuvant chemotherapy; GPRs: complete or ≥90% reduction of tumour cell mass; PPRs: no or ≤90% reduction of tumour cell mass; HFDs: healthy female donors; NS: nonsignificant; ^∗^statistically significant.

**Table 16:** Expression (%) of costimulatory and LNH molecules on mDC1s in the blood of women with LLABCs undergoing NAC. Baseline (B) levels in LLABCs versus completion of chemotherapy (CC) levels in different responders.

Study group comparisons		CD40	CD80	CD83	CD86	CD197
Good pathological responders (GPR: *n* = 9)	B (%)	40.60 ± 10.00	2.02 ± 1.30	4.45 ± 0.75	40.97 ± 14.00	31.29 ± 8.00
	CC (%)	43.13 ± 10.00	3.83 ± 2.00	3.71 ± 1.70	28.66 ± 7.00	48.34 ± 14.00
	B versus CC (*p* value)	NS	NS	NS	NS	NS
	CC versus HFDs (*p* value)	NS	NS	NS	NS	NS

Poor pathological responders (PPRs: *n* = 7)	B (%)	26.80 ± 10.00	1.48 ± 0.50	2.98 ± 2.00	21.37 ± 10.00	22.73 ± 6.00
	CC (%)	16.80 ± 8.00	2.29 ± 1.20	4.47 ± 1.80	6.81 ± 6.00	13.83 ± 5.00
	B versus CC (*p* value)	NS	NS	NS	NS	NS
	CC versus HFDs (*p* value)	0.001^∗^	NS	NS	0.001^∗^	NS

Post-NAC GPR versus PPR	GPR CC versus PPR CC (*p* value)	0.005^∗^	NS	NS	0.004^∗^	0.003^∗^

LNH: lymph node homing; LLABCs: large and locally advanced breast cancers; NAC: neoadjuvant chemotherapy; GPRs: complete or >90% reduction of tumour cell mass; PPRs: no or ≤90% reduction of tumour cell mass; HFDs: healthy female donors; NS: nonsignificant; ^∗^statistically significant.

## Abbreviations

A: Adriamycin
ALN: Axillary lymph node
C: Cyclophosphamide
CD: Cluster of differentiation
CTL: Cytotoxic T lymphocyte
DAB: Diaminobenzidine
DFS: Disease-free survival
DC: Dendritic cell
ER: Oestrogen receptor
FOXP3: Forkhead box protein 3
GPR: Good pathological response
HPF: High-power field
HRP: Horseradish peroxidase
IDO: Indoleamine 2,3-dioxygenase
IHC: Immunohistochemistry
IL: Interleukin
IFN-*γ*: Interferon-gamma
LLABC: Large and locally advanced breast cancer
MAb: Monoclonal antibody
mDC: Myeloid-derived dendritic cell
NAC: Neoadjuvant chemotherapy
NK: Natural killer
OS: Overall survival
pCR: Pathological complete response
pDC: Plasmacytoid dendritic cell
PMN: Polymorphonuclear neutrophil
PPR: Poor pathological response
RT: Room temperature
SLN: Sentinel lymph node
SOCS: Suppression of cytokine signalling
T: Docetaxel
TAA: Tumour-associated antigen
Th: T helper
TIN: Tumour-infiltrating neutrophil
TIM: Tumour-infiltrating macrophage
Treg: T regulatory cell
TGF-*β*: Transforming growth factor-beta
TIL: Tumour-infiltrating lymphocyte
VEGF: Vascular endothelial growth factor
X: Capecitabine.
