# Supplementary material for: The Differential Contribution of the Innate Immune System to a Good Pathological Response in the Breast and Axillary Lymph Nodes Induced by Neoadjuvant Chemotherapy in Women with Large and Locally Advanced Breast Cancers
Source: J Immunol Res. 2017 Aug 23;2017:1049023. doi: 10.1155/2017/1049023 (PMC5587972; doi:10.1155/2017/1049023)
Supplement: Supplementary file 1 — Table A. Patient and Tumour Characteristics, Responses to Neoadjuvant Chemotherapy (n=33). [file 1049023.f1.pdf]

Table A. Patient and Tumour Characteristics, Responses to Neoadjuvant Chemotherapy (n=33)

Patient No.	Age	BMI[1] (kg/m^2)	Menopausal Status[2]	Nodal Status (Clinical Assessment)	Tumour Size (mm)	Tumour Type	Histological Grade[3]	ER Status[4]	HER-2 Status[5]	NAC Regimen[6]	Clinical Response[7]	Pathological Response (Breast)[8]	Pathological Response (Axilla: Nodal Metastasis)[9]	Recurrence[10]	Death[10]
5	40	21.90	pre	+ve	40x50	ductal	3	+ve	-ve	4AC-4TX	responder	grade 5	grade 3	no	no
7	61	33.29	post	-ve	70x60	ductal	3	-ve	+ve	4AC-4T	responder	grade 5	NA	no	no
9	51	21.87	pre	-ve	34x32	ductal	3	-ve	-ve	4AC-4TX	responder	grade 5	NA	no	no
11	35	28.32	pre	+ve	100x82	ductal	2	+ve	-ve	4AC-4T	responder	grade 4	grade 3	yes	yes
12	57	24.44	post	+ve	47x42	ductal	3	+ve	-ve	4AC-4T	responder	grade 4	grade 2	yes	yes
13	43	31.56	pre	-ve	35x35	ductal	3	+ve	+ve	4AC-4T	responder	grade 3	NA	yes	no
15	35	25.85	pre	+ve	50x40	ductal	2	+ve	-ve	2AC-6TX	non-responder	grade 3	grade 2	no	no
17	61	23.81	post	-ve	36x21	ductal	2	+ve	+ve	4AC-4T	responder	grade 5	NA	no	no
20	57	26.29	post	-ve	38x35	ductal	2	+ve	-ve	2AC-6T	non-responder	grade 3	NA	no	no
21	48	30.25	pre	+ve	22x15	ductal	1	+ve	-ve	4AC-4T	responder	grade 3	grade 1	yes	no
22	47	23.52	pre	+ve	40x40	ductal	3	+ve	-ve	2AC-6TX	non-responder	grade 5	grade 3	no	no
29	54	25.49	post	+ve	34x27	lobular	2	+ve	-ve	4AC-4TX	responder	grade 3	grade 2	yes	no
37	52	24.70	post	-ve	40x39	ductal	1	+ve	-ve	4AC-4TX	responder	grade 3	NA	no	no
38	63	31.35	post	-ve	44x44	ductal	3	+ve	-ve	4AC-4T	responder	grade 4	NA	yes	yes
40	50	26.49	post	-ve	36x32	metaplastic	3	-ve	-ve	4AC-4T	responder	grade 5	NA	no	no
41	51	32.03	pre	-ve	30x30	ductal	3	+ve	-ve	4AC-4TX	responder	grade 5	NA	no	no
46	38	33.19	pre	+ve	39x38	ductal	3	-ve	+ve	4AC-4TX	responder	grade 5	grade 3	no	no
63	37	20.93	pre	+ve	35x30	ductal	2	-ve	+ve	4AC-4T	responder	grade 4	grade 2	yes	no
73	47	30.01	pre	+ve	33x33	ductal	3	-ve	+ve	4AC-4TX	responder	grade 5	grade 3	no	no
77	65	27.63	post	+ve	20x20	ductal	3	-ve	+ve	2AC-6T	non-responder	grade 1	grade 2	yes	yes
80	45	29.30	pre	+ve	36x31	ductal	2	+ve	-ve	2AC-6T	non-responder	grade 2	grade 1	yes	no
82	67	28.65	post	+ve	30x30	lobular	2	+ve	-ve	4AC-4TX	responder	grade 5	grade 2	no	no
83	50	22.37	pre	+ve	40x40	ductal	3	-ve	+ve	2AC-6TX	non-responder	grade 3	grade 2	no	no
84	47	26.29	post	+ve	45x30	ductal	2	+ve	-ve	4AC-4T	responder	grade 3	grade 2	yes	yes
86	64	32.37	post	-ve	40x40	ductal	2	+ve	-ve	2AC-6T	non-responder	grade 2	grade 2	no	no
88	58	32.97	post	+ve	25x15	ductal	2	+ve	-ve	2AC-6TX	non-responder	grade 4	grade 1	no	no
89	49	39.65	pre	+ve	25x25	ductal	2	+ve	-ve	2AC-6TX	non-responder	grade 2	grade 2	no	no
90	56	26.01	post	+ve	30x27	ductal	3	+ve	-ve	4AC-4TX	responder	grade 5	grade 3	no	no
96	51	25.28	post	+ve	45x45	ductal	3	-ve	-ve	2AC-6T	non-responder	grade 5	grade 2	yes	yes

108	56	24.92	post	+ve	47x40	ductal	3	-ve	+ve	4AC-4TX	responder	grade 5	grade 3	no	no
112	49	37.41	pre	+ve	50x40	ductal	2	+ve	-ve	4AC-4TX	responder	grade 5	grade 3	no	no
114	38	31.21	pre	+ve	42x36	ductal	3	-ve	+ve	4AC-4T	responder	grade 5	grade 3	no	no
115	65	32.22	post	+ve	14x12	ductal	3	+ve	-ve	4AC-4T	responder	grade 5	grade 3	no	no

(1) BMI: Body mass index (≤30: Non-obese, >30: Obese)

(2) Menopausal status: Pre-menopausal, age < 55 years with normal menstrual cycles; Post-menopausal, age > 50 years with no spontaneous menses for at least one year/ or age ≤ 50 years with no spontaneous menses within the past 2 years/or women who had bilateral oophorectomy prior to the diagnosis of breast cancer

(3) Histological grade: Grade 1 (well differentiated), grade 2 (moderately differentiated), grade 3 (poorly differentiated)

(4) ER (oestrogen receptor): Allred scoring system was used for measuring expression of ER (score ≥ 3 for positive, < 3 for negative)

(5) HER2 (human epidermal growth factor receptor 2): Determined by FISH (fluorescence in-situ hybridisation)

(6) A: Adriamycin (doxorubicin), C: Cyclophosphamide, T: Taxotere (docetaxel) and X: Xeloda® (capecitabine)

(7) Clinical response was assessed by MRI (magnetic resonance imaging) of breast after 2 cycles of AC using the RECIST criteria

(8) Pathological response in breast was graded as grade 1: No change or some alteration to individual malignant cells but no reduction in overall cellularity; grade 2: A minor loss of tumour cells but overall cellularity still high, up to 30 % loss; grade 3: Between an estimated 30% and 90% reduction in tumour cells; grade 4: A marked disappearance of tumour cells such that only small clusters or widely dispersed individual cells remain, more than 90% loss of tumour cells; grade 5: No malignant cells identifiable from the site of the tumour (pCR)

(9) Pathological response in axilla was graded as grade 1: Metastasis with no fibrosis; grade 2: Metastasis with variable replacement by fibrous tissue; grade 3: No malignant cells identifiable but replacement by fibrous tissue (pCR); NA (not applicable): No nodal metastasis

(10) Recurrent disease and death from a median follow-up of 51 months
